# Functional Evaluation in High Energy (Schatzker Type V and Type VI) Tibial Plateau Fractures Treated by Open Reduction and Internal Fixation

**DOI:** 10.1155/2014/589538

**Published:** 2014-10-30

**Authors:** Kavin Khatri, Devendra Lakhotia, Vijay Sharma, G. N. Kiran Kumar, Gaurav Sharma, Kamran Farooque

**Affiliations:** Department of Orthopaedics, Jai Prakash Narayan Apex Trauma Centre, All India Institute of Medical Sciences, Safdarjung Enclave New Delhi, Delhi 110029, India

## Abstract

*Objective*. To review functional outcome in high energy tibial plateau fractures treated by plating. *Design*. Retrospective analysis. *Material and Methods*. Sixty-five patients with Schatzker type V and type VI tibial plateau fractures treated with open reduction and internal fixation using plates were included in the study. The functional evaluation of the patients was carried out with Oxford knee scoring. *Results*. Fifty-four cases (83%) had Oxford knee score between 40 and 48. Seven (10.7%) had score between 30 and 39, three (4.6%) had score between 20 and 29, and one patient (1.5%) had a score of 18. Delayed union was seen in two cases and nonunion was seen in one case. The superficial wound infection was noticed in (9.2%) patients which was resolved with regular dressings and oral antibiotics. Three (4.6%) patients had developed deep wound infection and one among them had developed osteomyelitis. *Conclusion*. Open reduction and internal fixation in high energy tibial plateau fractures can provide good functional results in appropriately selected cases.

## 1. Introduction

Comminuted tibial plateau fractures were associated with functional disability [1]; however with the advent of various surgical devices there has been marked improvement in functional outcome. The goal in these types of fractures is to restore joint congruity, ensure joint stability, alignment, and achieve full range of motion [2]. However there are reports of disassociation between radiographic features and functional outcome. The articular incongruity may not be always correlated with poor functional outcome [3].

While treating these fractures not only the bony injury but also the soft tissue damage must be considered. Many authors have encountered poor results in patients treated with open reduction and internal fixation of tibial plateau fractures with poor soft tissue envelope [4].

The methods of fixation that have been used to address these fractures which include closed reduction and cast bracing [4, 5], open reduction and internal fixation [6], circular frame application [7–9], percutaneous screw fixation [10], ligamentosis, wire guided cannulated screw, minimal invasive plating techniques, and various other techniques are used with open approaches in conjugation with the open techniques to treat these fractures. Each technique has its own advantages and disadvantages.

High energy tibial fractures mostly affect the younger age group in productive life years and have significant socioeconomic impact due to time taken to recover and subsequent requirement of total knee replacement in some cases [11]. The specialisation in research has led to creation of various outcome measures like patient-, disease-, and domain-specific ones. These measures are more sensitive to change and useful in monitoring interventions. The outcome measures have moved towards functional limitations and complete body functions in order to assess an impact of the given condition on the activities in their specific environment. The aim of current study was to take such an approach to review the functional outcome in cases of Schatzker types V and VI tibial plateau fractures treated by open reduction and internal fixation.

## 2. Material and Methods

The study was conducted with the approval of ethics committee of the institution. The inpatient records of patients with Schatzker types V and VI tibial plateau fractures treated with open reduction and internal fixation between February 2009 and March 2013 were searched from medical record section of the institution.

One hundred and thirty-seven consecutive patients of tibial plateau fractures treated with open reduction and internal fixation were identified. Patients excluded from the study were tibial plateau fractures of Schatzker types I, II, III, and IV, open fractures, associated ipsilateral lower limb fractures, spine or pelvic fractures, and those lost to follow-up and treated by other methods.

Seventy-two patients were excluded from the study who did not meet the inclusion criteria. Five patients had died; sixty-seven patients could not be located or lost to follow-up. So, sixty-five patients (63 males and 2 females) were included in this study.

The hospital records included clinical history sheet and operative notes. They were studied to determine the mode of injury, demographic data, delay in surgery, treatment given, associated ligament injuries detected following fracture fixation, complications of either the fracture or treatment, and revision surgery if any required. The data regarding comorbid conditions, associated limb injuries, and side of injury was also collected. The surgery was delayed in cases with soft tissue injury indicated by soft tissue edema or blister formation. The patient was taken up for surgery once the soft tissue edema and blisters resolved with appearance of skin wrinkles.

**Figure 1:**
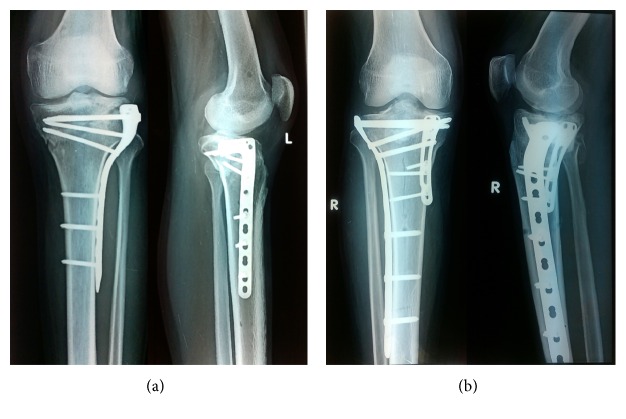
(a) Radiograph showing type V tibial plateau fracture treated with open reduction and internal fixation using lateral type locking plate. (b) Radiograph showing type VI tibial plateau fracture treated with open reduction and internal fixation using medial and lateral locking plates.

Preoperative antibiotic (1.5 gm cefuroxime intravenous) was administered in all the cases one hour prior to skin incision as a single dose. The operative procedures were performed in a standard operating room under regional or general anaesthesia under tourniquet control. The fracture was approached through anterolateral or posteromedial side or both depending upon the fracture configuration. The anterolateral exposure was performed with skin incision centring over Gerdy's tubercle. The posteromedial exposure was performed with skin incision posterior to the posteromedial edge of the tibia and extension along the course of pes anserinus tendons. Pes tendons were retracted anterior while medial gastrocnemius along with popliteus was retracted posteriorly to approach the posteromedial aspect of tibia. Anteromedial surface was dissected to minimum with limited subperiosteal elevation. Submeniscal arthrotomy was performed to visualize the articular surface and meniscal repair was done wherever required. Intraoperatively, fluoroscopy and direct visualization were used to achieve articular reduction. The fixed angle locking plates were used on the medial and lateral sides depending on the fracture configuration (Figures 1(a) and 1(b)). Medial proximal tibia plates are used to buttress the posteromedial fragment mainly which is often single and noncomminuted. The lateral proximal tibial plates are used in bridging mode; hence they are longer than the medial proximal tibial locking plates. Cannulated cancellous screw fixation was used wherever required. Subarticular defects were filled with autograft or bone graft substitutes in cases whenever required.

The radiographs including the immediate postinjury, postoperative, and follow-up radiographs were studied to confirm the correct classification of the fracture, treatment employed, articular reduction achieved, and any loss of articular reduction or malalignment on follow-up radiographs. Radiographs were assessed by all the authors to assess the articular reduction. The joint reduction was considered to be satisfactory if articular depression was less than or equal to five millimetres and plateau widening was less than or equal to five millimetres compared to the width of the distal femoral condyle. Computerized tomography was used to plan the treatment. Preinjury and postinjury employment status were also recorded.

The patients were advised non-weight-bearing mobilisation and quadriceps exercises on the first postoperative day. The patients were encouraged to do active assisted knee bending from third postoperative day. They were reviewed at 3 weeks, 6 weeks, 12 weeks, and at monthly intervals thereafter till radiological union and maximal functional recovery. Union was defined as bone healing by direct means as seen in at least two radiographic planes. Full painless weight bearing over the operated limb was considered as an indirect sign of healing. Subsequently they were followed at six-month intervals.

**Figure 2:**
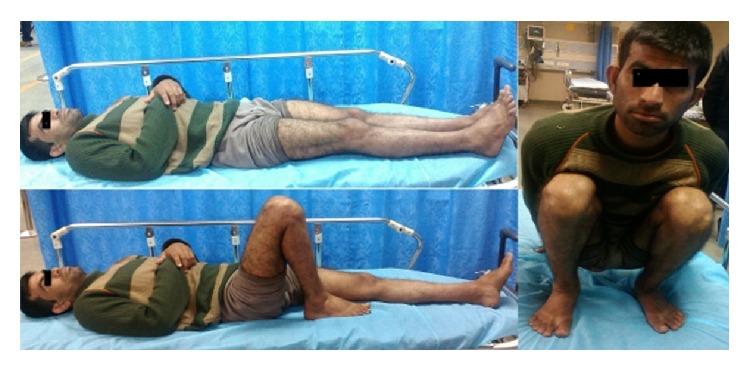
Image showing movement at knee joint in a high energy tibial plateau fracture (radiograph depicted as in Figure 1(a)).

All the patients were reviewed in the months of February and March 2014 with Oxford knee score (OKS) questionnaire [12]. The patients were asked twelve questions about the degree of pain in knee, any difficulty in toilet activities, ability to perform household activities, climbing up or down stairs, ability to knee, night pains, any limp in the operated limb, ability to kneel and stand again, and any discomfort in washing and drying oneself due to knee and various other questions mentioned in Oxford knee score. The maximum attainable score is 60. The patients were graded as poor (0 to 19), moderate (20 to 29), good (30 to 39), and excellent (40 to 48). The patients who had scored more than 40 were considered as cases with satisfactory functional outcome and minimal disability (Figure 2).

## 3. Statistical Analysis

Data was analyzed by using Student's *t-*test and correlations were analysed using the Pearson correlation coefficient. The statistical significance was determined at *P* value less than 0.05.

## 4. Results

The patients were followed up for duration ranging from 12 to 60 months. The mean duration of follow-up was 32 months. There were 27 tibial plateau fractures of Schatzker type V (41.53%) and 38 were of type VI (58.4%) treated by open reduction and internal fixation. The mechanism of injury was motor vehicle accident in 53 patients, pedestrian struck by vehicle in eight, falling from height in three, and history of assault in one case (Table 1). The age of the patients varied from 23 to 72 years (mean age = 42.98 years).

There was involvement of right side in 40 cases while the number was 25 on the left side and none of the cases had bilateral tibial plateau fracture. The majority of the fractures were treated within 14 days; however in five cases there was delay of more than two weeks. In two cases, there were multiple injuries and definitive fixation was staged while in the rest of the three patients surgery was delayed due to poor local skin condition.

There was loss of articular reduction manifested by articular depression (>4 mm) in five patients. Among these patients four had good functional recovery while one had fair outcome. None of the patients had tibial plateau widening greater than 5 mm with respect to femoral condyles. The mean medial proximal tibial angle was 84.2° (ranging from 83° to 92°) and mean posterior proximal tibial angle was 8.1° (ranging from 3° to 14°).

Two patients with closed fracture had undergone fasciotomy for the compartment syndrome and required skin grafting for the closure of wound. Three patients had ipsilateral peroneal nerve palsy and all of them had spontaneous recovery. Five patients had associated other musculoskeletal injuries including contralateral limb fractures and upper limb fractures. Eight patients (12.3%) had associated ipsilateral knee ligament and meniscal injuries detected after fixation of the fractures. Three among them had isolated medial collateral injury and one had associated lateral meniscus injury with medial collateral injury which was managed conservatively. Two patients had anterior cruciate ligament with lateral meniscus injury and one among them had medial collateral injury in addition to it. One patient had posterior ligament injury associated with medial meniscus injury. Ligament reconstruction was not done in these patients as they were not willing for the second operative intervention.

Superficial wound infection developed in six (9.2%) patients which was resolved with regular dressings and oral antibiotics. Three (4.6%) patients had developed deep wound infection and one among them had developed osteomyelitis. All of the three patients who had developed deep wound infection had to undergo secondary procedures. In two cases the debridement was done twice; however in one case even after three debridements there was no resolution of infection and decision to remove implant along with Ilizarov's fixation was carried out. Patient had developed nonunion in the follow-up period so secondary bone grafting with ipsilateral iliac crest being done at a later stage. One patient who had deep wound infection opted for implant removal after one year of surgery though there was no sign of infection at that time. Of the six patients who had developed infection, two had type 2 diabetes mellitus.

Delayed union was seen in two cases and nonunion was seen in one case. Primary bone grafting at the time of fracture fixation was carried out in eleven cases while secondary bone grafting was done in a case of nonunion. Of the eleven cases of primary bone grafting, bone graft substitutes in the form of calcium hydroxyapatite (G-bone, Surgiwear Pvt. Ltd., Shahjahanpur, India) were used in five cases while in the rest six cases and in case of secondary bone grafting ipsilateral iliac crest bone graft was used.

The functional outcome in our case series was assessed using Oxford knee score. Fifty-four cases (83%) had scored between 40 and 48. Seven (10.7%) had scored between 30 and 39, three (4.6%) had scored between 20 and 29, and one patient (1.5%) had a score of 18 (Table 2).

Age at the time of trauma had no correlation with the functional outcome (*r* = 0.0864; *P* = 0.4937) and moreover type of fracture (type V or VI) did not have any significant impact on the results (coefficient of determination = 0.0358; *P* = 0.777). The mean OKS score in type V was 43.962 and in type VI was 41.657. Infection had an impact on functional outcome in tibial plateau fractures (*t* = 6.36; *P* < 0.00001). The patients who experienced infection eventually had lower Oxford knee scores.

## 5. Discussion

The majority of the studies in orthopaedic literature with respect to operatively treated tibial plateau fractures are physician based assessments like radiographic articular reduction, motion at the knee, and any instability. The current trend is to use patient-specific tools to measure functional outcomes [13]. In our study OKS (Oxford knee score) was used for evaluation. It is a patient-specific tool to assess the patient's perspective regarding the outcome of the surgery. It was initially developed to assess the outcome following total knee arthroplasty, but subsequently its purview was extended to other surgical interventions around knee. It is a reliable, valid, simple, short, and practical questionnaire [14]. We had used one year as the minimum follow-up period because the results of knee are unlikely to change after one year and moreover function at the end of one year is a good prognostic indication for future knee function [15].

Open reduction and internal fixation with plating is considered one of the acceptable methods of treatment in Schatzker type V and type VI tibial plateau fractures. Excellent results in 81% of the cases have been reported in one of the series by Lachiewicz and Funcik [16]. Oh et al. [17] have also reported excellent results in 91% of the cases treated with open reduction and internal fixation of proximal tibial plateau fractures. Touliatos et al. [18] have reported excellent result in 57% of their cases while in our series 83% had excellent and 10.7% had good functional outcome. The comparison cannot be drawn between these studies as they have used different methods for evaluation of results.

Use of various methods of indirect reduction like Kirschner's wires and femoral distractor along with image intensifier was able to achieve acceptable articular reduction with minimal soft tissue damage. Hence early aggressive physiotherapy protocol could be started and excellent functional results were obtained.

The degenerative changes in knee joint are mainly due to inadequate restoration of articular surface, limb malalignment, joint instability, and delayed mobilization of knee joint [19]. However, Parkkinen et al. [20] in their study on lateral tibial fractures had reported that postoperative articular congruity and neutral mechanical axis had little effect on the medium term functional outcome in tibial plateau fractures. Marsh et al. [21] in their review of anatomic reduction in articular fractures had found little correlation between functional outcome and anatomical reduction. In our study also there was loss of articular reduction (>4 mm) in five patients. But four among these five had good functional recovery while one had fair outcome. Moreover, the patients in our study were mainly of middle age group (mean age—42 years) and did not experience new onset of osteoarthritis.

The reported incidence of soft tissue injuries associated with tibial plateau fractures injuries has been as high as 56% [22]. Stannard et al. [23] had reported association of ligament injuries in 85% of the cases of type VI while the number was 79% in type V tibial plateau fractures. They had also noticed high incidence of lateral meniscus injury in their study. However, Schatzker et al. [24] had observed the associated ligament injuries in 7.4% of the cases of the tibial plateau. Prasad et al. [12] had noticed anterior cruciate ligament injury in 6.5% of the cases and collateral ligament strain in 21% of the cases. In our study, 12.3% had associated ligamentous injuries. There is marked variation in the incidence of associated ligament injuries reported in the literature. The reason for that may be the use of magnetic resonance imaging by some authors (Stannard et al. [23]) while others (Schatzker et al. [24] and Prasad et al. [12]) have used clinical examination for the same to document the injuries. The soft tissue injuries can have marked effect on the functional outcome in tibial plateau fractures [25].

Tibial plateau fractures are fraught with various complications with respect to fixation methods and soft tissue envelope. There are numerous methods of management for the high energy proximal tibial fractures but the optimal treatment remains controversial. The high complication rate associated with open reduction and internal fixation was due to single midline incision that was used to approach both anterolateral and posteromedial fragments [26]. The dual incision to approach the posteromedial fragment and anterolateral fragment is associated with lower local wound complication [27].

**Figure 3:**
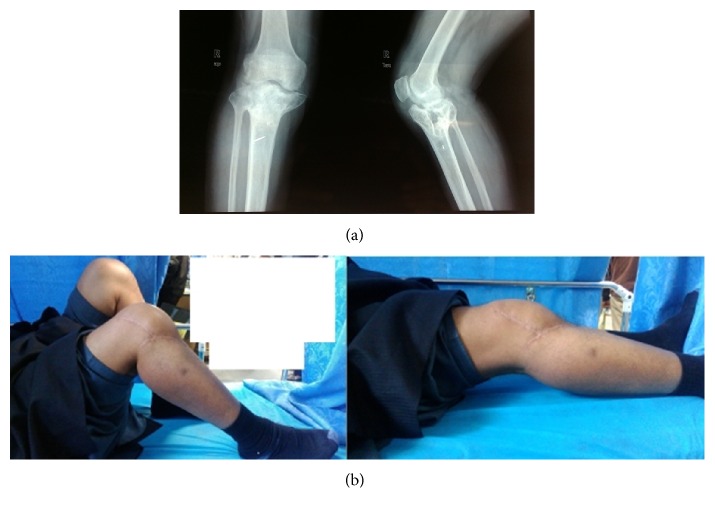
(a) Radiograph showing articular mal reduction, tibial condylar widening, and features of osteoarthritis. (b) Loss of movement at knee joint in a case who had developed deep infection.

Infection is one of the most dreaded complications encountered in the management of these fractures treated with open reduction and internal fixation. The timing of the surgery and careful handling of soft tissue along with minimal devascularisation can minimize the chances of infection [28]. Barei et al. [27] had reported deep wound infections (3.6%) in two patients and superficial infection in three patients (5.4%). Stevens et al. [29] reported one case of septic arthritis (2%) and five of superficial wound infections (10.6%) in their case series of 47 operatively treated patients. Nabil et al. [11] had reported infection in five of their 117 cases (4.2%), which were successfully treated with debridement and intravenous antibiotics. In our study, six patients (9.2%) had superficial infection. The infection was resolved with debridement and intravenous antibiotics. Three patients had developed deep wound infection and one among them had osteomyelitis that was treated by removal of hardware, debridement, and application of Ilizarov's external fixator along with autogenous bone grafting at later stage (Figures 3(a) and 3(b)). Lower Oxford knee scores were noted in patients who had infection (superficial or deep) as compared to those who did not experience any wound complication. Our results show that infection has an impact on functional outcome in tibial plateau fractures (*t* value = 6.36; *P* value is < 0.00001). The reason for it could be the decrease in compliance with the physiotherapy protocols in the patients who had infective episode. This may be either due to repeated surgeries or guarded approach in mobilising these patients.

Finally, we acknowledge that this study has certain limitations including lack of control group, follow-up period of less than five years, being not a single surgeon series, correlation between knee alignment and oxford knee scores being not ascertained, being not able to trace large number of patients, and retrospective nature of the study. Despite these limitations we believe this study provides reliable and valid information through the use of Oxford knee score and intermediate functional outcome in these types of injuries.

In conclusion, open reduction and internal fixation is an excellent method of treatment of type V and type VI tibial plateau fractures in judiciously selected cases. The high Oxford knee score in these cases supports the fact that operative intervention can alter the lifestyle of the patients markedly.

## Figures and Tables

**Table 1:** Patient data.

Case number	Age	Sex	Schatzker type	Associated injuries	Complication	Follow-up period (months)	Oxford knee score	Articular reduction
1	40	M	VI	Fracture multiple rib fractures on right side	None	26	47	Anatomic
2	41	M	VI	None	None	32	47	Anatomic
3	40	M	VI	None	None	12	44	Anatomic
4	44	M	VI	None	None	39	39	Nonanatomic
5	35	M	VI	None	None	24	45	Anatomic
6	55	M	VI	None	None	54	42	Anatomic
7	40	M	V	None	Superficial infection	18	36	Anatomic
8	47	M	VI	None	None	45	38	Anatomic
9	29	M	VI	None	None	14	35	Anatomic
10	52	M	VI	None	Deep infection, septic arthritis	59	18	Nonanatomic
11	31	M	V	None	None	25	46	Anatomic
12	41	M	V	None	None	18	44	Anatomic
13	32	M	VI	None	Superficial infection	34	47	Anatomic
14	28	M	VI	None	None	19	25	Anatomic
15	37	M	VI	None	Superficial infection	30	23	Anatomic
16	30	M	VI	None	None	13	46	Anatomic
17	35	F	V	None	None	25	44	Anatomic
18	62	M	VI	None	None	35	42	Anatomic
19	48	M	VI	None	None	36	43	Anatomic
20	45	M	VI	None	None	36	44	Anatomic
21	42	M	V	None	None	45	40	Nonanatomic
22	59	M	V	None	None	27	41	Anatomic
23	31	M	V	None	None	26	45	Anatomic
24	32	M	V	None	None	46	44	Anatomic
25	65	M	VI	None	Superficial infection	45	40	Nonanatomic
26	58	M	V	None	None	46	42	Anatomic
27	44	M	VI	None	None	44	45	Anatomic
28	30	M	V	None	None	34	43	Anatomic
29	42	M	V	None	None	13	44	Anatomic
30	35	M	V	None	None	14	42	Anatomic
31	60	M	VI	None	Superficial infection	22	39	Nonanatomic
32	49	M	VI	None	None	50	43	Anatomic
33	55	M	VI	None	Superficial infection	63	35	Anatomic
34	33	M	V	None	None	60	42	Anatomic
34	28	M	VI	None	None	60	44	Anatomic
35	47	M	VI	Fracture right capitellum humerus	None	59	42	Anatomic
36	38	M	VI	None	None	59	41	Anatomic
37	30	M	VI	None	None	50	43	Anatomic
38	72	M	VI	None	Late onset deep infection (10 months)	27	28	Anatomic
39	56	M	V	None	None	25	46	Anatomic
40	60	M	V	None	None	25	44	Anatomic
41	42	M	V	None	None	20	46	Anatomic
42	39	M	V	Fracture right proximal humerus	None	34	43	Anatomic
43	41	M	V	None	None	24	45	Anatomic
44	33	M	VI	None	None	14	46	Anatomic
45	57	M	VI	None	None	14	44	Anatomic
46	46	M	V	None	None	17	48	Anatomic
47	36	M	V	None	None	18	47	Anatomic
48	56	M	V	None	Deep infection	18	33	Anatomic
49	48	M	VI	None	None	18	47	Anatomic
50	40	M	VI	None	None	27	46	Anatomic
51	38	M	VI	None	None	24	46	Anatomic
52	35	M	V	None	None	24	47	Anatomic
53	32	M	VI	None	None	32	46	Anatomic
54	24	M	V	None	None	20	48	Anatomic
55	47	M	VI	None	None	45	46	Anatomic
56	25	M	V	None	None	46	46	Anatomic
57	53	M	VI	None	None	35	48	Anatomic
58	60	F	VI	None	None	57	46	Anatomic
59	39	M	VI	None	None	56	49	Anatomic
60	59	M	VI	None	None	47	48	Anatomic
61	23	M	V	Fracture neck of left scapula and fracture midshaft clavicle	None	19	47	Anatomic
62	46	M	V	None	None	19	46	Anatomic
63	45	M	V	Fracture right distal end radius with left shaft femur	None	12	48	Anatomic
64	52	M	VI	None	None	12	46	Anatomic

**Table 2:** Functional outcome in terms of Oxford knee score.

Oxford knee score	Number of patients
0–19	1
20–29	3
30–39	7
40–48	54

Total	65
